# Exosomes from COVID-19 Patients Carry Tenascin-C and Fibrinogen-β in Triggering Inflammatory Signals in Cells of Distant Organ

**DOI:** 10.3390/ijms22063184

**Published:** 2021-03-20

**Authors:** Subhayan Sur, Mousumi Khatun, Robert Steele, T. Scott Isbell, Ranjit Ray, Ratna B. Ray

**Affiliations:** 1 Department of Pathology, Saint Louis University, St. Louis, MO 63104, USA; subhayan.sur@health.slu.edu (S.S.); mousumi.khatun@health.slu.edu (M.K.); Robert.steele@health.slu.edu (R.S.); scott.isbell@health.slu.edu (T.S.I.); 2 Department of Internal Medicine, Saint Louis University, St. Louis, MO 63104, USA; rayr@slu.edu

**Keywords:** COVID-19, exosomes, mass spectrometry, tenascin-C, fibrinogen-β, cytokines, pathogenesis

## Abstract

SARS-CoV-2 infection can cause cytokine storm and may overshoot immunity in humans; however, it remains to be determined whether virus-induced soluble mediators from infected cells are carried by exosomes as vehicles to distant organs and cause tissue damage in COVID-19 patients. We took an unbiased proteomic approach for analyses of exosomes isolated from plasma of healthy volunteers and COVID-19 patients. Our results revealed that tenascin-C (TNC) and fibrinogen-β (FGB) are highly abundant in exosomes from COVID-19 patients’ plasma compared with that of healthy normal controls. Since TNC and FGB stimulate pro-inflammatory cytokines via the Nuclear factor-κB (NF-κB) pathway, we examined the status of tumor necrosis factor-α (TNF-α), interleukin-6 (IL-6), and C–C motif chemokine ligand 5 (CCL5) expression upon exposure of hepatocytes to exosomes from COVID-19 patients and observed significant increase compared with that from healthy subjects. Together, our results demonstrate that TNC and FGB are transported through plasma exosomes and potentially trigger pro-inflammatory cytokine signaling in cells of distant organ.

## 1. Introduction

Emergence of a novel severe acute respiratory syndrome coronavirus 2 (SARS-CoV-2) has precipitated the current global health crisis, with many deaths worldwide. The World Health Organization announced COVID-19 to be a Public Health Emergency of International Concern and then declared COVID-19 as a pandemic. SARS-CoV-2 is an enveloped virus containing a 29.9 kb positive-sense RNA genome. The virus genome contains at least ten open reading frames (ORFs). The first ORF (ORF1a/b), representing about two-thirds of the viral RNA, is translated into two large polyproteins, which are processed into 16 non-structural proteins (nsp1-nsp16), and some of them form the viral replicase transcriptase complex [1]. The other ORFs of the SARS-CoV-2 genome encode four main structural proteins: spike (S), envelope (E), nucleocapsid (N), membrane (M), and several accessory proteins of unknown functions.

The lung is a vital organ that supports blood oxygenation and decarboxylation necessary for aerobic life. Any insult, including viral infection, may impair this process and compromise survival. Lung immune responses and inflammatory processes are tightly regulated to maintain respiratory function. The lungs are highly susceptible to developing innate immune responses to viral infection, such as SARS-CoV-2. Although the viral envelop protein interacts with angiotensin-converting enzyme 2 (ACE2) present on many cell surfaces as a receptor, lung epithelial cells are probably the most susceptible cells for SARS-CoV-2 entry and replication causing human disease. Clinical observations indicate that severely ill COVID-19 patients develop extrapulmonary tissue/organ dysfunctions, although viremia is not common. The presence of SARS-CoV-2 RNA in blood was reported in very low number of postmortem samples [2] and these observations are debatable [3]. The pathophysiology of extrapulmonary manifestations is not entirely clear, but thought to be due in part to a dysregulated inflammatory response characterized by inhibition of interferon signaling by the virus, T cell lymphodepletion, and the production of pro-inflammatory cytokines, particularly interleukin-6 (IL-6) and tumor necrosis factor-alpha (TNF-α) [4,5]. Extrapulmonary manifestations of SARS-CoV-2-associated disease have been documented in numerous organ systems including, but not limited to, cardiac, neurologic, hemostatic, kidney, and liver.

Exosomes (30–150 nm) are extracellular vesicles that play an important role in intercellular communication by inducing physiological changes in recipient cells through the transfer of bioactive lipids, nucleic acids, and proteins. Exosome vesicles are formed by the interior budding of endosomal membranes to form large multivesicular bodies (MVBs). Exosomes play an important role in cellular homeostasis and in the pathogenesis of major human diseases. Evidence suggested that exosomes carry materials from one cell to other cells for initiation and exaggeration of disease [6]. Exosomes are also involved in viral spread, immune regulation, and antiviral response during infection [7,8,9]. Little is known about exosomes of SARS-CoV-2-infected patients and their role in pathogenesis. In this study, we observed that exosomes from the plasma of COVID-19 patients harbor tenascin-C and fibrinogen-β, and trigger inflammatory signal in distant cells.

## 2. Results

### 2.1. Isolation and Characterization of Exosomes from COVID-19 Patient Plasma

**Figure 1:**
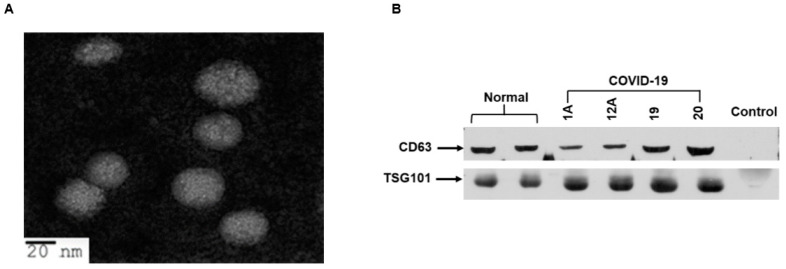
Transmission electron microscopy and characterization of exosomes from plasma of COVID-19 patients. (**A**) Representative transmission electron microscopy (TEM) image of exosomes isolated from COVID-19 patient plasma. The sample grids were screened under a JEOL JEM-1400Plus transmission electron microscope. The exosomes were round in shape with diameters of 30–70 nm. (**B**) Exosome lysates from plasma of normal and COVID-19 patients were subjected to Western blot analysis for detection of CD63 and TSG101 protein using specific antibodies. A representative image shows results from normal and COVID-19 exosomes (1A, 12A, 19, and 20). Exosome depleted serum was used as a negative control.

Patient information and samples used in this study are shown in Table 1. In this small cohort, we had twenty COVID-19 patients’ plasma who were admitted to the ICU in our academic medical center, and the samples were collected that day. Most of the plasma samples (15/16) had higher D-dimer values and four samples’ values were unknown. We isolated exosomes from the plasma of 20 COVID-19 patients and 8 healthy volunteers (normal). The size and purity of exosomes were examined by transmission electron microscopy (TEM) and observed spheres of heterogeneous size (30–70 nm particles) (Figure 1A). The presence of exosomal markers, CD63 and TSG101, was verified from the exosome preparations by Western blot analysis as described previously [10,11] and the results from a representative blot is shown (Figure 1B). CD63 and TSG101 are commonly used markers for exosomes and are present in exosomes isolated from serum, plasma, and body fluids. Exosome depleted serum (Invitrogen) was used as a negative control and, as expected, did not exhibit the presence or cross-reactivity for CD63 or TSG101 proteins. We further examined whether SARS-CoV-2 RNA was present in exosomes. For this, we used the primer sets that recognize SARS-CoV-2 nucleocapsid gene using a Centers for Disease Control (CDC)-recommended PCR kit (10006770 from IDT-2019-nCoV_N1: targets virus nucleocapsid (N) gene for specific detection of SARS-CoV-2, 2019-nCoV_N2: targets virus nucleocapsid (N) gene for specific detection of SARS-CoV-2, and RP: targets human RNase P gene for detection of human nucleic acids; control for sample integrity). SARS-CoV-2 RNA was undetected in exosome preparations from patients or healthy subjects.

### 2.2. Exosomes from Plasma of COVID-19 Patients Harbor Tenascin-C and Fibrinogen-Β

**Figure 2:**
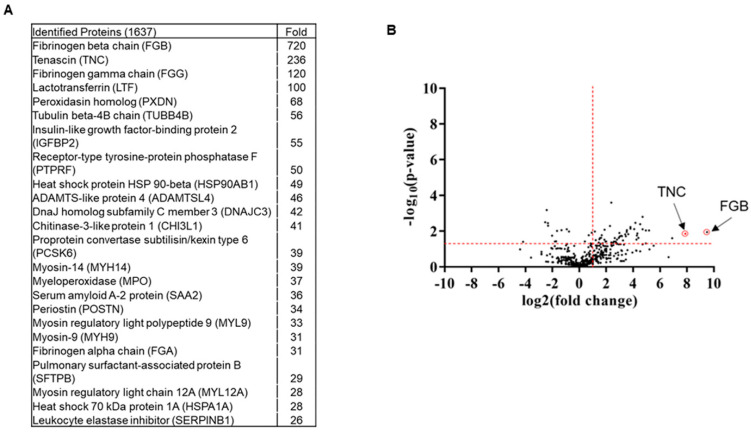
Comprehensive changes in plasma proteome profile of COVID-19 patients. (**A**) Top hits of COVID-19 exosomal proteins compared with normal exosomes are shown as a fold increase. (**B**) Volcano plot illustrates significant difference in the fold change of proteins in COVID-19 exosomes compared with normal exosomes. The x-axis represents log2 (fold change) and the y-axis is log10 (*p*-value) showing statistical significance. Horizontal dashed red-line showing *p* = 0.05 (−log_10_(0.05) = 1.3) and vertical dashed red line represents fold change (COVID/normal exosomes) at 2 (log_2_(2) = 1). The absolute fold change and *p*-value 0.05 were considered as the threshold cut-off. TNC and FGB are shown in red circles.

Little is known about the proteome profile of exosomes from COVID-19 patients. Using an unbiased proteomic approach, mass spectrometry analysis identified 1637 proteins. We shortlisted 163 proteins having more than five spectra counts and at least twofold changes compared with plasma exosomes from healthy volunteers (Figure 2A,B). Tenascin-C (TNC) and fibrinogen-β (FGB) were identified as the two significantly enriched molecules in exosomes from COVID-19 patients relative to normal exosomes.

**Figure 3:**
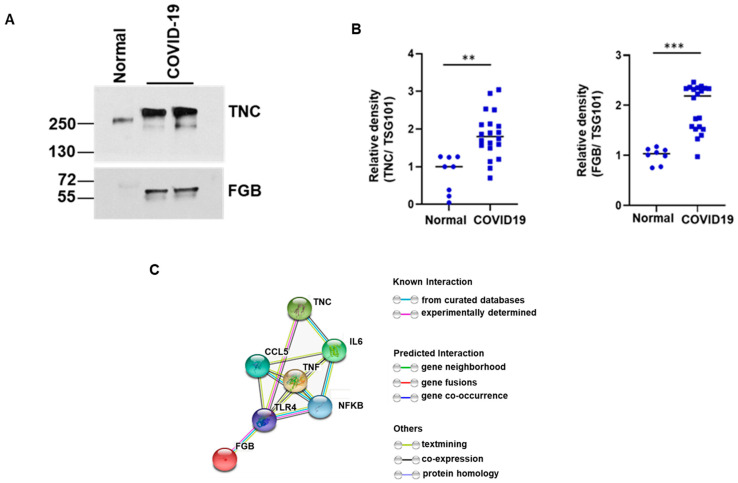
Tenascin-C (TNC) and fibrinogen-β (FGB) are highly present in exosomes of COVID-19 patients. (**A**) Lysates from COVID-19 plasma exosomes and normal exosomes were subjected to Western blot analysis for TNC and FGB using specific antibodies and a representative image is shown. (**B**) Dot plots for quantitative Western blot band intensities by densitometry analysis using ImageJ software are shown (*n* = 8 normal and *n* = 20 COVID-19 samples). TSG101, an exosomal marker protein, was used for normalization of each sample. (** *p* < 0.01; *** *p* < 0.001). (**C**) String analysis network module represents functional association of TNC and FGB with TLR4/NF-κB signaling. Each node represents all the proteins produced by a single protein coding gene. Colored node represents query proteins and first shell of interactions. Filled node shows 3D structure (known or predicted). Edges represent protein–protein associations for shared function.

TNC is an immunomodulatory hexameric extracellular matrix glycoprotein that induces chronic inflammation and fibrosis in organs, including lung, liver, and kidney, by interaction with toll-like receptor 4 (TLR4) and integrin receptors [12]. However, association of TNC with SARS-CoV-2 infection was unknown. FGB is one of the components of the fibrinogen complex cleaved by the protease thrombin into fibrin to form blood clots [13,14]. Increased levels of blood fibrinogen and associated disorders, such as coagulopathy and venous thromboembolism, are observed in COVID-19 patients [15]. Enhanced TNC and FGB expression in exosomes isolated from COVID-19 patients was verified by Western blot analysis (Figure 3A). The antibody from Sigma (AB19011) recognizes TNC around 250–350 kDa polypeptides. We also noticed a difference in the molecular size pattern in the control lane. This could be due to the recognition of different glycosylated forms. We further examined the expression of TNC and FGB in the exosomes of all COVID-19 and control samples. The expression of these proteins in the exosomes from normal plasma was significantly lower compared with exosomes from COVID-19 patients (Figure 3B). Acute inflammation is characterized by increased production of cytokines, and the primary feature of COVID-19 pathology is severe lung injury and multi-organ failure [12,16,17]. We performed a protein–protein interaction (PPI) network analysis of TNC and FGB using STRING to study the potential interactions between them. As shown in Figure 3C, the PPI network diagram contains several proteins interacting with TNC. FGB and TNC induce pro-inflammatory cytokine production through interaction with the inflammatory NF-κB signaling pathway. These results suggest that COVID-19 patient plasma exosomes harbor TNC and FGB and transport them to distant organs for virus associated pathogenesis.

### 2.3. Exosomes Isolated from COVID-19 Plasma Trigger Pro-Inflammatory Cytokines in Hepatocytes Through Activation of NF-κB Signaling

**Figure 4:**
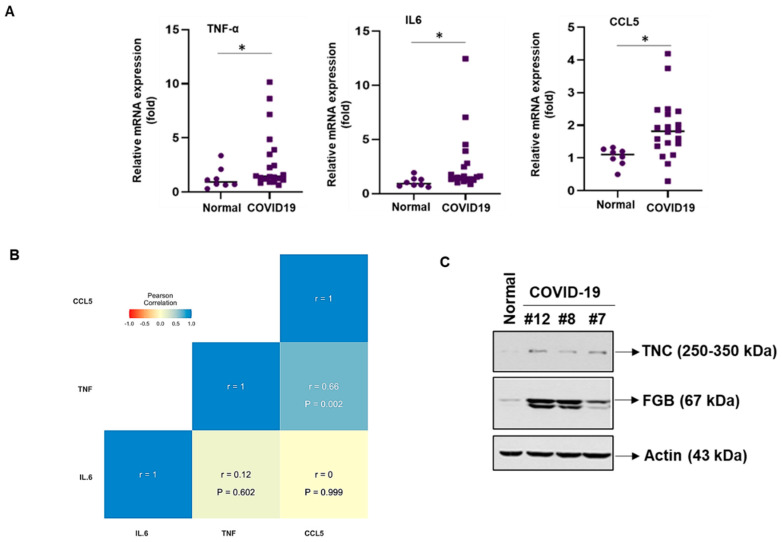
Exposure of hepatocytes to exosomes from COVID-19 plasma triggers pro-inflammatory molecules. (**A**) Immortalized human hepatocytes (IHHs) were exposed to normal and COVID-19 exosomes for 48 h and total RNA was isolated. Relative mRNA expression of tumor necrosis factor-α (TNF-α), interleukin-6 (IL-6), and C–C motif chemokine ligand 5 (CCL5) in cells treated with COVID-19 exosomes (*n* = 20) or normal exosomes (*n* = 8) were examined by qRT-PCR and represented by dot plots. We used 18s rRNA as an internal control. Small bar indicates standard error (* *p* < 0.05). (**B**) Pearson correlation analysis among expressions of the TNF-α, IL-6, and CCL5 in the hepatocytes exposed with patient exosomes. (**c**) Huh7 cells were exposed with normal and COVID-19 exosomes for 48 h and cell lysates were subjected to Western blot analysis for TNC or FGB using specific antibodies. The membrane was reprobed for actin as an internal control.

To investigate the association of patient exosomes with inflammation, hepatocytes were used as a model cell line and exposed to exosomes from either COVID-19 patients or healthy controls. Significant upregulation of tumor necrosis factor-α (TNF-α), interleukin-6 (IL-6), and C–C motif chemokine ligand 5 (CCL5) was observed from exposure of immortalized human hepatocytes (IHHs) to exosomes isolated from COVID-19 patient plasma compared with exosomes from healthy normal plasma (Figure 4A). Similar results were noted from COVID-19 plasma exosomes when exposed to a different cell line of hepatocyte origin (Huh7). We computed the Pearson’s correlation coefficients among expressions of the TNF-α, IL-6, and CCL5 in the hepatocytes exposed to patient exosomes. A significant positive correlation (P = 0.002, r = 0.66) was seen between TNF-α and CCL5 expression from hepatocytes (Figure 4B). This suggested that TNF-α and CCL5 values increase concurrently. Further, we did not observe the presence of these molecules in exosomes from either normal subjects or COVID-19 patients. To gain insight into the exosome-mediated uptake of TNC/FGB in hepatocytes, we examined the presence of TNC/FGB from exosome-treated hepatocyte lysates by Western blot analysis. We observed the higher presence of TNC/FGB in COVID-19 exosome-treated hepatocytes compared with normal exosome-treated hepatocytes (Figure 4C), verifying the exosome-mediated uptake of TNC/FGB by hepatocytes. These data suggested that TNC and FGB are the potential inducers for inflammation in hepatocytes and further examination is necessary for a correlation and causation. Together, our results demonstrated that COVID-19 plasma exosomes trigger strong pro-inflammatory cytokine production in hepatocytes.

**Figure 5:**
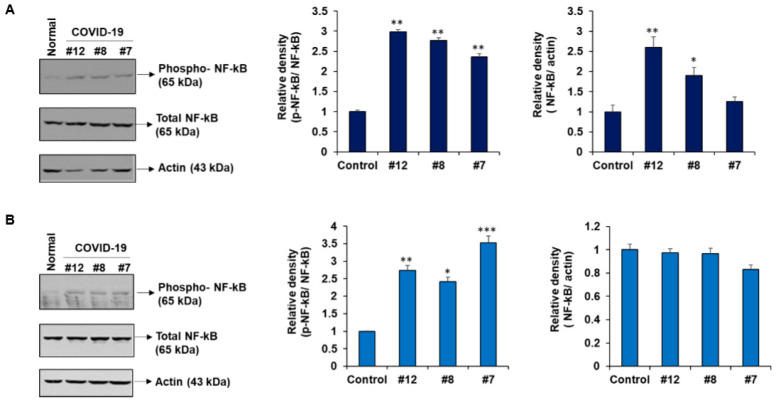
Exosomes from plasma of COVID-19 patients activates NF-κB signaling in hepatocytes. IHH (**A**) or Huh7 cells (**B**) were exposed to normal and COVID-19 exosomes for 48 h and cell lysates were subjected to Western blot analysis for phospho-NF-κB p65 (Ser536), NF-κB p65 using specific antibodies. The membrane was reprobed for actin as an internal control. The same actin blot from Huh7 cells is used in Figure 4C. The right panel shows quantitative representation of Western blot band intensities. Small bar indicates standard error (* *p* < 0.05; ** *p* < 0.01; *** *p* < 0.001).

NF-κB is a key regulator of inflammation, innate and adaptive immunity, proliferation, and cell survival [18]. We observed a significant increase in phospho-NF-κB upon exposure of hepatocytes (IHH or Huh7) to exosomes from COVID-19 patients compared with that from normal plasma (Figure 5A,B). This result further demonstrates that exosomes from COVID-19 plasma, enriched with TNC and FGB, generate cytokine production by activation of NF-κB signaling in hepatocytes.

## 3. Discussion

The novel observations from this study reveal: (i) elevated levels of TNC and FGB in exosomes from plasma of COVID-19 patients and (ii) COVID-19 exosomes enhance expression of pro-inflammatory cytokines TNF-α, IL-6, and chemokine CCL5 through the NF-κB signaling pathway upon exposure to hepatocytes, as a model for cells from distant organs. Our results explain a different trans-regulatory mechanism for multiorgan pathogenic disorders during SARS-CoV-2 infection.

An array of clinical studies demonstrated that COVID-19 patients experience a cytokine storm [17,19], although specific consequences and related mechanisms are not well-defined. There are conflicting reports on the presence of SARS-CoV-2 RNA in patient blood, but we failed to detect viral RNA in the exosomes, and a similar observation was reported recently [3,20]. Hijacking the exosomal pathway by several RNA viruses has been demonstrated to mediate endogenous intercellular communication, immune modulation, and pathogenesis [6,9,21]. We and others have shown that exosomes from HCV infected hepatocytes carry messages for activation of hepatic stellate cells and induce fibrosis marker expression [11,22]. GM3-enriched exosomes in COVID-19 patients are reported using lipidomics [23], although the functional consequence is yet to be determined.

We focused here on the inflammatory molecules since they play a major role in COVID-19-related pathogenesis. The association of TNC with SARS-CoV-2 infection has not been previously reported. TNC induces chronic inflammation and fibrosis via an interaction with toll-like receptor 4 and integrin receptors. Elevated expression of TNC in exosomes is reported in glioblastoma patients and in nasal lavage fluid during human rhinoviruses infection [12,24]. Increased levels of blood fibrinogen and venous thromboembolism are reported in COVID-19 patients [15]. In addition, FGB is involved in other disease processes, such as wound healing, liver injury, allergic airway disease, cardiovascular disease, and microbial pathogenesis by modulating the host immune system. FGB is primarily synthesized in hepatocytes, although extrahepatic epithelial cells also synthesize fibrinogen [25]. Elevated FGB expression was reported in lung adenocarcinoma, suggesting a potential role as a biomarker [26]. Elevated fibrinogen is detected in exosomes from drug- and alcohol-induced liver injury, neurological disorder, and swine flu viral infection [14,27,28,29,30]. 

**Figure 6:**
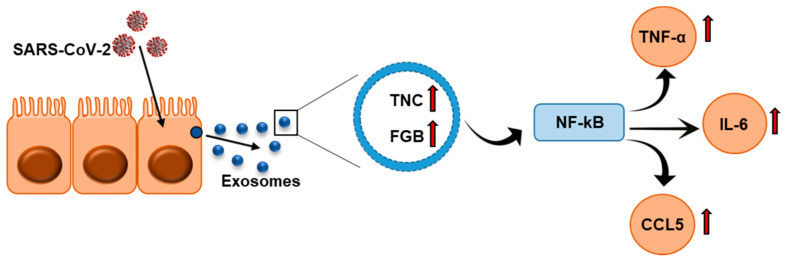
Schematic representation shows exosomes secreted from SARS-CoV-2-infected cells are enriched with TNC and FGB triggering TNF-α, IL-6, and CCL5 production in hepatocytes via NF-κB signaling.

Chronic inflammation and increased cytokine production, like TNF-α, IL-6, and CCL5, are critical features of COVID-19 patients, which suggests that they cause severe lung injury, multi-organ failure, and poor prognosis [16,17,31,32,33]. We observed activation of NF-κB signaling and activation of target genes, TNF-α, IL-6, and CCL5, following exposure of hepatocytes to exosomes isolated from plasma of COVID-19 patients (Figure 6). In SARS-CoV2 infection, increased levels of IL-6 and STAT3 are reported [31,34]. The spike protein of SARS-CoV2 triggers IL-6 production [5]. Analyzing SARS-CoV-2 genetic materials in exosomes may be important for understanding whether viral genetic materials play a role in cytokine induction in distant organs. Other molecules present in exosomes from the plasma of COVID-19 patients may also have a role in disease processes and need to be evaluated in future studies. Several studies suggest that non-coding RNAs carried through the exosomes play a crucial role in virus-mediated disease progression and warrant investigation. The presence of TNC and FGB in SARS-CoV-2-infected patient exosomes may act as potential modulators for the induction of inflammatory cytokines, resulting in microthrombosis in some patients. The exosomes used in this study were isolated from patients admitted to the ICU in our academic medical center. Analyzing exosomes from SARS-CoV-2-infected patients with mild symptoms in future will help with understanding whether TNC and/or FGB can be used as potential prognostic markers. Further, how TNC and FGB are enhanced in COVID-19 patients will be important to understand. In conclusion, our results suggested that exosomes carry TNC and FGB in hospitalized COVID-19 patients, and exposure of cells from distant organs may trigger cytokine expression. Our work also highlighted for the first time that TNC- and FGB-enriched exosomes from COVID-19 plasma may be correlated with pathogenesis.

## 4. Materials and Methods

### 4.1. Plasma Specimens

A total of 20 deidentified, heparinized plasma specimens, collected from COVID-19 patients admitted at the Saint Louis University Hospital, were used. All patients were confirmed positive for SARS-CoV-2 by RT-PCR performed on a nasopharyngeal swab around the time of hospital admission. Patient specimens were collected for clinical laboratory analyses as part of routine clinical care. Patient information is summarized in Table 1. This study was waived by the Saint Louis University Institutional Review Board for use of deidentified clinical specimens. These samples were kept frozen at −70 °C. Archived plasma samples from eight healthy volunteers were included as normal control and were collected from the pre-COVID-19 era for a different study.

### 4.2. Exosome Isolation and Analysis

Exosomes were isolated from plasma using the ME Kit (ME-020p-Kit) following the supplier’s instruction (New England Peptide Inc, Boston, MA, USA). The exosomes were examined after negative staining using a JEOL JEM-1400Plus transmission electron microscope.

### 4.3. Cell Culture and Exposure with Exosomes

Immortalized human hepatocytes (IHHs) and a human hepatoma cell line (Huh7) were maintained in Dulbecco’s modified Eagle’s medium (DMEM) supplemented with 10% fetal bovine serum (FBS) and 1% penicillin/streptomycin at 37 °C in a 5% CO_2_ atmosphere. IHH and Huh7 cells were seeded into 6-well plate at a density of 3 × 10^5^ cells/well and exposed to equal concentration of exosomes for 48 h. Cells were harvested for RNA or protein analyses.

### 4.4. Mass Spectrometry Analysis

The exosome pellet was dissolved in lysis buffer (4% SDS, 100 mM dithiothreitol (DTT), 150 mM Tris–HCl pH 8.0) and was subjected to mass spectrometric analysis using a Thermo Q-Exactive system. Peptides were separated on an EASYnLC system with a Thermo ES803 PepMap C18 column. The results were acquired in data-dependent acquisition mode; top10 m/z for MS2 per cycle (Washington University Proteomics Shared Resource). Candidate proteins were defined as those having a minimum 5 spectra count and at least 2-fold enrichment compared with normal.

### 4.5. RNA Isolation and Analysis

Total RNA was isolated from exosomes or hepatocytes (IHH or Huh7) for qRT-PCR as described previously [11] using TaqMan Universal PCR master mix and 6-carboxyfluorescein (FAM)-MGB probes for SARS-CoV-2 [2019-nCoV CDC EUA kit (10006770, IDT)], CCL5 (assay ID: HS009822282_m1), IL-6 (assay ID: HS00985639_m1), and TNF-α: (assay ID: HS00174128_m1) following the manufacturer’s protocol (Thermo Fisher Scientific, Berkley, MO, USA). We used the 18s rRNA (assay ID: Hs03928985_g1) as the endogenous control. The relative gene expression was analyzed using the 2^−∆∆CT^ formula (ΔΔ*C_T_* = Δ*C_T_* of the sample −Δ*C_T_* of the control). Each sample was loaded in triplicate for analysis.

### 4.6. Western Blot Analysis

Cell lysates were subjected to Western blot analysis using specific antibodies to CD63 (Santa Cruz Biotechnology, Dallas, Tx, USA), TSG101 (Santa Cruz Biotechnology), tenascin (TNC) (Sigma), fibrinogen-β (FGB) (Santa Cruz Biotechnology), phospho-NF-κB p65 (Ser536) (Cell Signaling Technology, CST, Denvers, MA, USA), and NF-κB p65 (CST). The blot was reprobed with actin-horseradish peroxidase (HRP) antibody (Santa Cruz Biotechnology) to compare protein load in each lane. Densitometry analysis was done using ImageJ software (National Institutes of Health, Bethesda, MD).

### 4.7. Statistical Analysis

The results are expressed as mean ± standard error. Student’s *t*-test was used for comparison between two groups (normal vs. COVID-19 exosomes). Pearson’s correlation analysis was performed using GraphPad Prism software. *p*-values of < 0.05 were considered statistically significant. All experiments were repeated at least three times, and representative data are shown.

## Figures and Tables

**Table 1:** Sample information from COVID-19 patients.

Parameters		Number
**COVID-19 patients**		20
**Age**	>60 years	11
	<60 years	9
**Sex**	Male	8
	Female	12
**D-dimer**	<0.5 µg/mL FEU	1
	>0.5 µg/mL FEU	15
	Unknown	4

FEU: fibrinogen equivalent unit.

## Data Availability

The data generated for this study are included in this manuscript.
